# Cubosomes: Design, Development, and Tumor-Targeted Drug Delivery Applications

**DOI:** 10.3390/polym14153118

**Published:** 2022-07-31

**Authors:** Hassaan Umar, Habibah A. Wahab, Amirah Mohd Gazzali, Hafsa Tahir, Waqas Ahmad

**Affiliations:** 1 School of Pharmaceutical Science, Universiti Sains Malaysia, Minden 11800, Malaysia; hassaanumar@student.usm.my (H.U.); amirahmg@usm.my (A.M.G.); 2 Department of Nutrition Sciences, University of Management and Technology, Punjab 54770, Pakistan; hafsa.tahir@umt.edu.pk

**Keywords:** cubosomes, glyceryl monooleate, drug delivery, active targeting, passive targeting

## Abstract

Because of the extraordinary advancements in biomedical nanotechnology over the last few decades, traditional drug delivery systems have been transformed into smart drug delivery systems that respond to stimuli. These well-defined nanoplatforms can boost therapeutic targeting efficacy while reducing the side effects/toxicities of payloads, which are crucial variables for enhancing patient compliance by responding to specific internal or external triggers. Cubosomes are lipid-based nano systems that are analogous to well-known vesicular systems, such as lipo- and niosomes. They could be used as part of a unique drug delivery system that includes hydro-, lipo-, and amphiphilic drug molecules. In this review, we critically analyze the relevant literature on cubosomesregarding theories of cubosomeself-assembly, composition, and manufacturing methods, with an emphasis on tumor-targeted drug delivery applications. Due to the bioadhesive and -compatible nature of cubosome dispersion, this review also focuses on a variety of drug delivery applications, including oral, ophthalmic and transdermal.

## 1. Introduction

The loss or diminution of cellular control and normal maturation pathways is a hallmark of cancer. Excessive cell development, undifferentiated cells and tissues, and the capacity to expand into surrounding tissues and metastasis are all characteristics of cancer. The excision of tumor and possibly neighboring tissues, chemo- and immunotherapy, radiation treatment, or combination of these are all options for treatment. However, some treatments have been linked to serious side effects. As a result, one of the most active fields of cancer research is the development of systems that might selectively deliver medication molecules to the diseased site without increasing their levels in the healthy parts of the organism [1]. A current trend in nanomedicine and drug delivery research is studies on improving the specificity of the treatment to increase efficacy and avoid side effects. Nanoparticles have played a large role in this trend because they can accumulate in target tissues either passively (via the so-called enhanced permeation and retention effect [2]) or actively (using surface-conjugated targeting ligands [3]).

Targeted drug delivery, also known as “drug targeting”, is used frequently and is quite different from “targeted therapy”, which is used in drug discovery [4]. Drug targeting can be defined as the accumulation of certain amounts of drugs at the specific or targeted site regardless of the route and method of drug administration. Targeted therapy is quite different and involves the interaction of the moiety with its receptors at a molecular level. Four components play integral roles in effective drug targeting, namely retain, evade, target, and release [5]. For intravenous administration, the drug is loaded to an efficient carrier for delivery, remains in circulation for sufficient time period, is retained within intended sites, and then is released for the effective functioning of the drug [6].

Drug targeting can be distinguished by two types, namely active and passive. These terms are usually used for in vitro analysis and do not correspond to the in vivo drug behavior. Passive targeting refers to the collection of the drug around the leaky vasculature of tumor sites, which is called the enhanced permeation and retention (EPR) effect. Passive targeting happens to almost all drug carriers, regard less if such targeting is required or not. The bulk (>95%) of the dispensed nanoparticles is believed to aggregate in the liver, spleen, and lungs; however, the EPR effect can be attained for the intravenous administration of nanoparticles. So, when >95% of an administered dose of a drug ends up in non-specific sites of the body, it cannot be called selective targeting. As such, it is evident that passive targeting is inappropriate—it is delivery of the drug by blood circulation in the body. Therefore, it should be replaced by the term extravasation or blood circulation because it is not selective and occurs after the majority of the drug administration process has concluded. Efficient “blood circulation and extravasation” is evident in locally activated delivery, where the drug remains at a specific site, such as around the tumor but not in other parts of the body [7].

“Active targeting” is specifically known as a ligand–receptor interaction and can also be narrated as the interaction of a drug/drug vector with the intended cells. Ligand–receptor interactions can only occur when the two moieties are in close proximity (<0.5 nm). The term “active targeting” resembles a cruise missile guiding the moiety to a specific target compared with traditional drug delivery systems, where the drug is mainly distributed by circulation and is retained at unintended sites [8,9,10]. Active targeting is a specific “ligand–receptor-type interaction” for intracellular localization. So, in order to achieve active (or effective) targeting, there should be enhanced blood circulation. PEGylation (the modification of nanoparticles with poly (ethylene glycol)) or enhancing the EPR effect can extend blood circulation time. The results also show that the presence of a tumor-targeting ligand does not necessarily lead to greater nanovector retention in tumors, implying that “active targeting” does not always imply “effective delivery to the entire tumor” [11,12]. Moreover, pegylated cubosomes exhibited an improved targeting of certain aggressive tumors, i.e., ovarian cancer cells [13].

**Figure 1:**
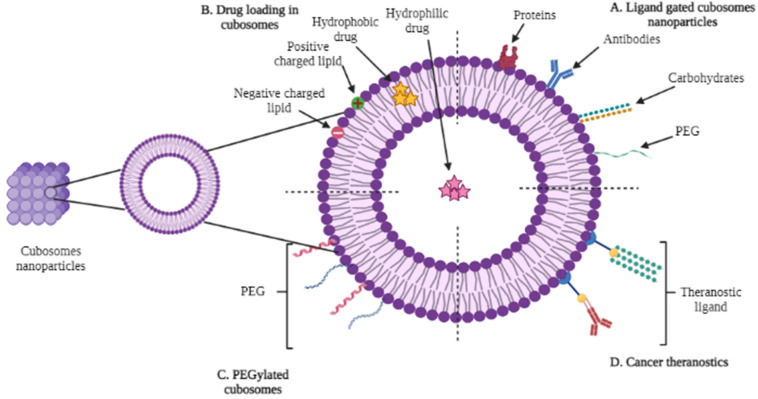
Cubosomes exhibiting internal and cubic structures with potential of drug delivery.

The drug delivery facilitated by the lipid nanoparticle has proven fundamental in the development of liquid crystalline colloidal carriers, e.g., cubo-, spongo-, and hexosomes, and vesicles as indicated in Figure 1 [14]. Cubosomes are bi-continuous lipids containing nanoparticles, and have discrete, sub-micron, cubic liquid crystalline phases. The formulation of these nanocarriers involves a specific ratio of certain surfactants in order to form fine dispersion [15]. Cubosomes are nanoparticles or, more precisely, nanostructure particles, which are generated by the self-assembly of amphiphilic- or surfactant-like molecules in liquid crystalline phases with cubic crystallographic symmetry. Surfactant-controlled cubosome structuresare separated by two zones of water, and are also optically isotropic, viscous, and characterized by a solid lipid crystalline cubic symmetry that is comparable to honeycomb (cavernous) structures, and range in sizes from 100 to 500 nm. The continuous repeating and sophisticated 3D structures of cubic liquid crystalline phases provide excellent opportunities to entrap hydrophobic, hydrophilic, and amphiphilic components [16]. Bi-continuous water and oil channels are formed by amphiphilic molecules, with bi-continuous referring to two distinct (but non-intersecting) hydrophilic regions separated by the bilayer. The structure’s interconnectivity produces a clear, viscous gel with rheology, which looks similar to cross-linked polymer hydrogels [17].

The structure entirely distinguished cubosomes from hexosomes, which contains hexagons due to its inverse hexagonal phase. The cubosome assemblies present further advantages because the transformations between the different liquid crystalline organizations, e.g., Pn3m, Im3m, and Ia3d, in addition to the inverted hexagonal phases, can be tuned and controlled by changes in the temperature, ionic strength, or pH of the environment of the targeted application sites [18,19].

Cubosomes are isotropic and transparent in nature, and their cubic liquid crystals are physically stable when in excess of water. They are biodegradable, non-toxic, and have the ability to solubilize hydrophobic, hydrophilic, and amphiphilic compounds. Due to their thermodynamic stability, cubosomes last indefinitely [20]. Colloidal dispersions of cubosomes can be stabilized by the addition of polymers. They also possess the potential for the controlled delivery of actives, where diffusion is governed by the tortuous diffusion of the active through the “regular” channel structure of the cubic phase [21]. Due to their narrow pore sizes, cubosomes facilitate the controlled release of drugs and they are vital for maintaining the stability and efficacy of biologically active compounds, such as proteins and vitamins [22]. Compared with other liquid crystalline drug delivery systems, such as lipo- and hexosomes, cubosomes have lower viscosity and exist at any dilution in water and further cubic phases [23,24].

The cubic structure of cubosomes has the ability to entrap the moiety and then release agents based on different molecular weights and polarities, which follows the laws of Higuchi-diffusion-controlled kinetics [25,26].

(1) $$Q=\left[D_{m}C_{d}\left(2A-Cd\right)t\right]½$$

According to equation, the release (diffusion) of agents from the matrix depends on the square root of time. *Q* is the quantity of the agents released per unit area of the matrix, where *D_m_* is the diffusion coefficient of the agents in the cubic matrix, *C_d_* is the solubility of the agents in matrix, *A* is the primary quantity of the drug per unit volume of the matrix, and *t* is the time. From that equation, the quantity and rate of drug release can be estimated.

Nanostructured cubosomes, made by fragmenting bulk cubic phase gels or by lyotropic techniques, have the same cubic phase inner-structure but a substantially larger specific surface area and reduced viscosity. Cubosomes are regarded as promising vehicles for various routes of drug administration due to their unique properties, such as thermodynamic stability, bioadhesion, their ability to encapsulate hydrophilic, hydrophobic, and amphiphilic substances, and their potential for controlled release through functionalization. This review introduces cubosomes, concentrating on their structures, preparation methods, potential administration routes, and tumor-targeted drug delivery based on the most recent publications.

## 2. Types of Cubosomes

Cubosomes can be differentiated into liquid and powdered cubosome precursors depending upon the method used for formulation. Cubosomes can be spontaneously formed by the dilution of monoolein with a hydrotropicsolvent, such as ethanol. The nucleation process permits particles to form, which increases through crystallization and precipitation processes. The quid precursor technique allows for a faster cubosome preparation scale-up while avoiding bulk solids handling and possibly destructive high-energy operations. This method could be applied to produce cubosomes in situ [17].

In addition to the liquid cubosome precursors, powdered cubosomes can be prepared using dehydrated surface-active agents that are incorporated with a suitable polymer. By using the spray-drying technique, the powdered cubosomes can be formed after the encapsulation of particles from liquid droplets in emulsion and dispersion. This technique utilizes the coating of a waxy lipid on water-soluble and non-cohesive starch, which usually prevents agglomeration [27]. However, it is better to use cubosomes in a dry powder rather than liquid form in order to prevent the processing of bulk water.

## 3. Components of Cubosomes

Amphiphilic lipids and stabilizers of various grades are the main components of cubosomes Table 1. Lipids are used to formulate cubic crystals based on their amphiphilic profile and these include monoglycerides, glycol- and phospholipids, and alkyl glycerates. Depending on the composition, lipid molecular structure, electrostatic interactions, and pressure and temperature, these amphiphilic lipids can self-assemble and form a wide range of nanostructures with distinct physicochemical properties and geometries [28].

The most common and widely used amphiphilic lipid for the formulation of cubosomes is glyceryl monooleate (GMO), which is also referred to as monoolein. The physical and chemical properties of GMO, which are significant for cubosome synthesis, include the polarity of the unsaturated monoglyceride, which has a melting point of 35–37 °C, storage temperature of 20–30 °C, and a HLB value of three, and it is usually colorless and clear in nature. In order to make bi-continuous cubic structures, GMO can be synthesized by a mixture of glycerides and estersfrom oleic and other fatty acids and mainly consists of monooleate [37].

**Figure 2:**
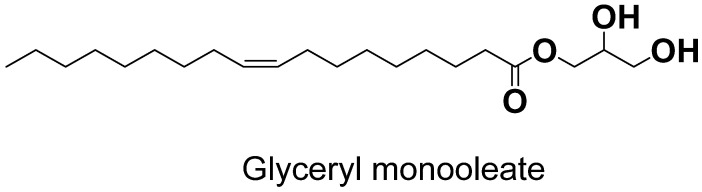
Molecular structure of GMO.

Due to presence of hydroxyl groups in the head portion of the GMO structure, which contributed to the formation of a H-bond with water molecules and hydrocarbon chains in the tail, GMO acts as an amphiphile because it has hydrophilic and -phobic characteristics (Figure 2). In 1984, it was first recommended as a biocompatible encapsulating material. Being non-toxic and biocompatible and -degradable, it is used as an emulsifier in the food industry and in many pharmaceutical preparations [38].

**Figure 3:**
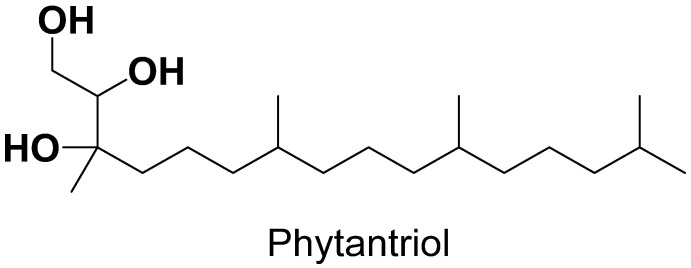
Structure of phytantriol.

Along with GMO, phytantriol is the other lipid used in cubosome formulation due to its increased penetration power and emulsification properties. Compared with monoglycerides, phytantriol gained more interest recently because of its characteristics, including better chemical stability due to absence of an ester group (Figure 3) and an improved moisture retention. Commercially available phytantriol has high purity (95%), while monoglycerides are present with different purities because they are synthesized from various sources. PHYT-based liquid crystalline matrices are considered a remarkable drug delivery system because they are able to release drugs at sustained rates, especially hydrophilic moieties [30,39].

Cubosomes are composed of bulk cubic aggregates that are thermodynamically stable but they are not kinetically stable due to the interaction of the hydrophobic portion with external hydrophilic aqueous media. So, in order to increase the stability of the formulation, there is a need to use a stabilizer in order to avoid the agglomeration of the dispersed particles into the parent bulk cubic structure. The stabilizer provides the electrostatic barrier between the particles in order to keep them dispersed and to avoid re-coalescence [40].

The stabilizer affects the dispersed particles’ structures and controls their phase behaviors. In particular, a sufficient concentration of P407 ensures the presence of the P-type cubic phase, which is in charge of forming a stable colloidal dispersion. The internal liquid crystalline structure and chemical makeup determine the frequency and nature of interactions between P407 and bulk liquid crystalline systems. For instance, P407 is integrated into the liquid crystalline structure of the GMO cubic phase as opposed to being adsorbed onto the surface of the PHYT cubic phase [41].

Pluronics are the most widely used stabilizing agents in the formation of cubosomes, especially the F127 (gold standard), which is also known as “Poloxamer 407”. These copolymers are composed of polyethylene oxide (PEO) and polypropylene (PPO) and are water soluble. The hydrophobic and -philic properties of Pluronics are because of the polypropylene and -ethylene oxide portions of Pluronics. The stabilizer can be used in different concentrations depending on the dispersed particles and types of lipids used in the formulation. In preparations containing GMO as a lipid, Pluronics are added up to 20% *w*/*w* depending on the total weight of the dispersion and the concentration of the stabilizer varies from 2.5% to 10% (*w*/*w*) [42].

The use of non-ionic molecules as a stabilizer has been popularized recently instead of using F127. The researchers found that the stabilizing effect of the stearate class of poly (ethylene oxide) is more effective than the F127 when used in the formulation of phytantriolcubosomes. In particular, product Myrj59^®^, which is 100 poly (ethylene oxide) units, was more effective in stabilizing phytantriolcubosomes when compared with the gold standard F127. The mechanism and reason for this enhanced stability are still unclear. On the other hand, polyvinyl alcohol (PVA) also showed great results in stabilizing the dispersion, and it can be used in cubosome preparations [33].

## 4. Methods of Preparation for Cubosomes

Techniques to form cubosomesinclude the bottom-up, top-down, spray-drying, and solvent evaporation methods. However, the main approaches for preparation are the top-down and bottom-up techniques. Both techniques require a suitable stabilizer, such as F127, to prevent cubosome dispersion aggregation, as previously described. The selection of the optimum method is to ensure stability, biocompatibility, and optimal drug release through the cubic matrix of the cubosomes [43].

### 4.1. Top-Down Approach

This is the most widely used method in the synthesis of cubosomes, especially when glyceryl monooleate (GMO) is used as a lipid polymer. This technique requires the application of high levels of energy in order prepare the fine dispersion of cubosomes. The steps involved are: first form the bulk cubic aggregates by adding a suitable stabilizer to a lipid; then, apply high energy through a homogenizer in order to form the dispersion. Fortunately, cubosomes prepared by this technique are stable for up to one year. However, the main drawback of this method is that when preparing large-scale batches, the application of high levels of energy is not viable when temperature-sensitive bioactive agents, especially peptides and proteins, are incorporated [44].

### 4.2. Bottom-Up Approach

The other method forcubosome preparation is the bottom-up approach, which is widely used when preparing cubosomes containing phytantriol as the lipid. This approach is also known as the solvent dilution method. This method requires a low energy input compared withthe top-down approach. This approach focused on forming cubosomes with a hydrotrope and stabilizer in an excess of water by applying minimal energy. In this technique, the hydrotrope is the key factor, as it controls the solubilization processto dissolve the water-insoluble lipids. It also prevents the formation of liquid crystals at high concentrations. The hydrotrope acts as a surface-active agent and is a molecule able to solubilize poorly soluble agents in aqueous media by hydrotropic solubilization. The bottom-up technique has advantages over the top-down approach because it requires low energy; thus, it can be safely used for the preparation of cubosomes incorporated with temperature-sensitive agents. Due to the uniform dispersion of stabilizers on the surface of the produced nanoparticles, this technique shows long-term formulation stability [45,46].

### 4.3. Spray-Drying Method

The spray-drying method is used in order to formulate powder precursors of cubosomes, and involves the use of organic solvents that evaporate upon the application of air. The lipid–surfactant solvent mixture is atomized with a wave of hot air, resulting in rapid solvent evaporation and the formation of the dry powder form of the cubosome precursor. It starts with mixing the lipid with the stabilizer and then dissolving them into ethanol (binary solutions can also be used). A separate mixture of aqueous phase, consisting of a hydrophilic solid carrier, i.e., sorbitol or dextran, is prepared, which is then combined with the previously prepared lipid mixture. Both mixtures are combined with continuous stirring, which leads to the formulation of a low viscosity emulsion. This technique is simple, cost effective, and normally easy to scale-up [47].

### 4.4. Solvent Evaporation Method

Solvent evaporation is another method of producing powder cubosomes by using a homogenizer or ultra-sonicator. The process is quite similar to the spray-drying method, except for the use of a high-energy sonicator. In this method, first, an organic solvent such as ethanol or chloroform is used to dissolve the lipids, which are then added dropwise to the other mixture containing a stabilizer, such as Pluronics inaqueous phase. The mixture is maintained at an elevated temperature under magnetic stirring. The drug can be dispersed in the lipid or the aqueous surfactant solution. Stirring under elevated temperatures removes the volatile organic solvent, and the mixture is homogenized by ultra-sonication or homogenizer, resulting in the formation of cubosomes [48]. Table 2 illustrates the benefits and drawbacks of these four techniques, which are usually used in the formation of cubosomes.

## 5. Physical–Chemical Characterizationsof Cubosomes

The physical and chemical characterizations of cubosomes include the evaluation of morphology using scanning electron or transmission electron microscopes. The particle size of a nanocarrier can be tested using dynamic light scattering (DLS). It also includes the evaluation of the zeta potential of the nanocarrier, and the polydispersity index (PDI) and drug encapsulation efficiency (EE%) of cubosomes. Table 3 describes the physical and chemical characterization of cubosomes. 

## 6. Cubosomes as Tumor-Targeted Drug Delivery

Cancer is a huge threat to human life. Even after 50 years of research, the standard conventional treatment is not always effective in treating the disease. Only lymphocytic leukemia and Hodgkin’s lymphoma can be successfully treated with conventional therapy. The main drawback of conventional chemotherapy is that it delivers the toxic anticancer agent indiscriminately to tumors and normal organs and tissues. So, standard treatment isalso harmful to normal cells and tissues in the body. Therefore, there is a need to formulate a therapy that only targets the cancerous site, and does not affect the normal healthy tissues. This can only be established by delivering the antitumor drugs selectively to affected areas, thereby protecting the normal tissues. Exploiting the structural and pathophysiological anomalies of tumor tissues, particularly the tumor vasculature, and employing the increased permeability and retention (EPR) effect, is one of the most successful drug delivery techniques [57].

Anticancer drugs have a restricted clinical use due to their non-specific distribution in living creatures, thereby causing side effects in normal tissues. So, in order to overcome this, various drug carriers have been developed to deliver agents at specific sites. Nanocarriers are among the most effective to target the disease site, and is based on the enhanced permeation and retention effect (EPR effect). Targeting ligands that are complementary to specific or overexpressed receptors on cancer cells, for example, has been utilized to functionalize carrier surfaces, and an external magnetic field can also help in tumor medication delivery. The tumor microenvironment should be taken into consideration because it is heterogeneous in nature. Due to high interstitial pressures and a thick extracellular matrix (ECM), the delivery by nanoparticles can be compromised. However, by changing the size and improvising the microenvironment of the cancerous site, the accumulation and penetration of a nanocarrier can be enhanced. By normalization of tumor vasculature and deforming the ECM, the reflux of nanoparticles can be reduced, leading to high concentrations of the carrier at a specific tumor site. However, there are certain unanticipated side effects that should be considered regarding the disruption of ECM, which can enhance tumor migration and ultimately lead to tumor metastasis. The premature restoration of blood arteries may make it harder for nanoparticles to passively target tumor tissues via the EPR effect. The extent and timing of the microenvironment modification must be carefully balanced [58,59].

In the late 1960s, the concept of a bi-continuous liquid crystal phase was originally presented, and it was finally realized in the mid-1970s. The reverse bi-continuous cubic phases, especially those based on the monoolein–water system, are the most studied and fascinating of these phases. The applications of modern pharmaceutical nanotechnology in a variety of pharmaceutical sectors, including enzymes, antimuscarinic medicines, antibiotics, and analgesic administration, have been thoroughly researched and reviewed, thanks to the rapid advancement of modern pharmaceutical nanotechnology. The cubic liquid crystal, which could be diffused in water, has good biocompatibility and -adhesivity. Because of their promising qualities, these multifunctional delivery systems can be administered in a variety of methods, including intravenous, oral, percutaneous, and ocular injections. This new cancer treatment, which involves specifically delivering anticancer medicines to the tumor site while minimizing their buildup in healthy organs, has been hailed as a potential technique for reducing treatment side effects [60]. So, we provide a detailed review, which highlights the prospects of cubosomesbeing used in targeted drug delivery to various tumors Table 4.

### 6.1. Skin Cancer Therapy

Paclitaxel (PTX), a model medication, was encapsulated in this study using monoolein-based cubosomesstabilized by Pluronic F127 and polyethylene glycol polymers. The study was carried out to evaluate the in vivo tumor growth inhibition by paclitaxel (PTX)-loaded cubosomes (CB), and for that purpose, A431 tumor-bearing mice were randomly divided into three groups (five mice per group): Group 1—PBS control; Group 2—PTX control; and Group 3—PTX-CB. We administered 120 μL of the corresponding formulations, which contained a dose of PTX per 15 mg/kg body weight, to each animal. The study revealed that PTX inhibited the A431 tumor growth by lowering the tumor volume of 250 mm^3^ compared with 360 mm^3^ in the untreated PBS group after two weeks of treatment. Mice treated with PTX-loaded cubosomes lowered the tumor volume of 160 mm^3^, which exhibited a 0.7-fold reduction in the final tumor volume compared withthe PTX-free group. This might be due to the whole-body biodistribution study’s findings that PTX-CB accumulates preferentially at tumor locations compared with PTX-free [61].

Another study revealed there was an active targeting of cancer cells by incorporating paclitaxel (PX) to cubosomes that were designed to be monoolein-based nanocarriers. To do this, biotin, a targeted ligand with an affinity for the sodium-dependent multivitamin transporter (SMVT) that is overexpressed in the membrane of tumor cells, was coupled with PF108, a surfactant typically employed to maintain cubosomes. Biotinylated cubosomes with a high level of functionally active biotin on their surface significantly improved the anticancer activity of PX at a concentration of 1 µg/mL when compared with PX or the nontargeted cubosomes. Additionally, the biotin ligand encourages receptor-mediated endocytosis, which enhances the cancercell uptake of the formulation. These findings imply that the biotinylated-cubosome-targeting technique can lower PX toxicity while increasing PX efficacy against tumor cells [62].

### 6.2. Glioblastoma Multiforme Therapy

Utilizing a top-down method, glyceryl monooleate (GMO) and the surfactant Pluronic F-127 were used to create cubosomes that were loaded with AT101. Because of its capacity to accelerate apoptosis in tumor cells by autophagic cell death, AT101, the R-(-)-enantiomer of the cotton-seed-derived polyphenol gossypol, is a potential medication in glioblastoma multiforme (GBM) therapy. The main drawback of the drug is its low solubility in aqueous solutions and its low bioavailability, which hinder its response during treatment. To circumvent this limitation, AT101 is loaded on cubosomes to increase bioavailability and improve AT101’s anticancer capabilities. Cubosomes, as AT101 drug carriers in GBM cells, are first-time prepared and found to be effective in treatment [63]. The advantage of employing cubosomes while delivering hydrophobic moiety, such as AT101, is an enhancement in the drug’s bioavailability due to its greater solubility in the lipid membrane of the produced nanoparticles, and ultimately their dispersion in water-based media. The pronounced internalization linked to endocytic pathways may also play a role in the enhanced cytotoxicity response to GMO-AT101 cubosomes. Proteins with high binding affinities for AT101 molecules display lower AT101 activity. AT101 is unable to attach to the proteins in the culture medium due to cubosome-based carriers.

### 6.3. Lung Cancer Treatment

Inhalation medication delivery is extremely advantageous for treating non-small-cell lung cancer (NSCLC) because it requires less dosages and reduces systemic toxicity. An FDA-approved antituberculosis medicine called bedaquiline (BQ) has previously demonstrated outstanding anticancer activity. However, its transport via the lungs is constrained by weak water solubility. BQ-loaded cubosome (BQLC) nanocarriers were formulated that can be inhaled to treat NSCLC. The cubosomal nanocarriers used in the preparation of the BQLC had a particle size of 150.2 ± 5.1 nm, a zeta potential of (*+*) 35.4 ± 2.3 mV, and an encapsulation efficiency of 51.85 ± 4.83%. After 48 h of treatment, the BQLC showed an improved cellular internalization and cytotoxicity with a 3-fold lower IC_50_ compared with free BQ in NSCLC (A549) cells. The BQLC decreased colony growth and cancer spread in vitro by suppressing cell proliferation via the apoptotic route [51].

### 6.4. Colorectal Cancer Therapy

Cisplatin- and metformin-loaded cubosomes were prepared using GMO, F-127, and polyvinyl alcohol and tested against colorectal cancer. Colorectal cancer (CRC) is a dreadful tumor and remains a leading cause of death worldwide. Cisplatin has been found to be successful in treating cancer, although it is associated with serious side effects and drug resistance. Combining anticancer drug treatment with effective drug carriers is needed. That is why cisplatin and metformin are incorporated in the cubosomes formulation. When compared with unformulated cisplatin, cubosomal formulations had a stronger cytotoxic effect. During the treatment of HCT-116, the inhibitory concentration (IC50) of cisplatin was found to be 15 µM, while cisplatin-loaded nano cubosomes reduced the IC50 to 9.6 µM. Furthermore, including metformin into the cisplatin-loaded cubosomes reduced the IC30 cisplatin to 7 µM, inhibiting cell growth by 50%. Cisplatin-loaded nano cubosomes caused glucose and energy levels to drop, resulting in adenosine-monophosphate-activated protein kinase (AMPK) activation and mammalian target of rapamycin (mTOR) suppression. The aforementioned formulation resulted in a significant increase in ROS levels, as well as an increase in NADPH oxidase and caspase 3, and the inhibition of LDH. The cytotoxic effects of the drug-loaded nanoparticles were larger than the effects of the individual drug, as evidenced by the fraction of the cell survival values [64,65].

### 6.5. Liver Cancer Treatment

In the presence of Poloxamer 407 as a stabilizer, a cubic gel phase of monoolein and water was disrupted to create cubosomal dispersions. The goal of the study was to encapsulate the anticancer medication 5-fluorouracil (5-FU) in order to combat liver cancer. The monoolein and water cubosomal dispersions were made with Poloxamer 407 as a stabilizer. On formulations containing 5-FU, in vitro and in vivo experiments were conducted. The entrapment efficiency was determined to be 31.21% for the drug when compared with a 5-FU solution, revealing nanometer-sized particles with a narrow particle-size distribution. During the first hour of drug release from the cubosomes, nearly half of the entrapped drug was rapidly released, followed by a slower drug release. Rats given 5-FU-loaded cubosomes displayed severe dilatation and congestion to the central (CV) and portal veins (PV) and hepatic sinusoids (S). Additionally, it was discovered that some of the hepatocytes had fatty alterations, and inflammatory cells had infiltrated the space between the hepatocytes in the portal area. The free-5-FU-treated group displayed only minor congestion in the central and portal veins along with fatty alterations and ballooning degeneration in the hepatocytes at the periphery of the hepatic parenchyma, as opposed to the effects of 5-FU-loaded cubosomes. This pattern shows that the free 5-FU group has less hepatotoxicity than the 5-FU-loaded cubosome group. Lower dosages of 5-FU in cubosomal formulations for the treatment of hepatocellular carcinoma need to be further studied for hepatotoxicity and in vivo anticancer efficacy [66].

### 6.6. Ovarian Cancer Treatment

Icariin was loaded on cubosomes comprising GMO and P407 as a stabilizer. The potential of icariin (ICA)-loaded cubosomes in treating ovarian cancer was explored. The Box–Behnken statistical design was used to optimize the cubosome formulation. The drug entrapment efficiency of the formulations ranged from 78.3 to 97.3%, with particle sizes ranging from 73 to 183 nm. Against ovarian cancer cell lines (SKOV-3 and Caov 3), optimized ICA-loaded cubosomes (ICA-Cubs) showed better cytotoxicity and apoptotic capacity than ICA-raw. The optimized ICA-Cubs were found to be non-toxic to EA. hy926 endothelial cells. The analysis of cell cycle arrest showed a promising role for optimized cubs in the pre-G1 and G2/M phases in comparison with ICA-raw. The enhanced levels of the ICA’s apoptotic potential may be associated with this increased creation of ROS. Therefore, it is anticipated that treatment with ICA-Cubs has a stronger ability to decrease TNF production within the cytosol of the SKOV-3 cells, which may interfere with angiogenesis in the tumor microenvironment, as well as the growth, proliferation, invasion, and metastasis of cancer cells. The formulation strategy has a substantially greater impact because ICA-Cubs produce significantly more caspase-3 than ICA-raw. The enhanced internalization of ICA in the novel formulation may be the cause of this; the placebo formulation had no effect on the amount of caspase-3 in ovarian cancer cells. This function of caspase-3 in the therapy group may be related to p53 expression, which may have an impact on cancer cell growth and metastasis. Due to enhanced drug solubility and cellular permeability, it was concluded that ICA loaded cubosomes have the potential to suppress ovarian cancer cell development [67].

### 6.7. Cervical Carcinoma

Temozolomide and doxorubicin were mounted on cubosomes carrying miR-7-5p and comprising monoolein and F108. The effects of drug- and miRNA-loaded vehicles were investigated using molecular biology techniques, such as quantitative real-time PCR, anMTS-based cell proliferation test, flow cytometry, and spheroids formation assay. The anticancer effects were tested using cervical carcinoma-derived (HeLa) cells. MiR-7-5p enhances cell sensitivity to medicine, and nanoparticles containing both miRNA and pharmaceuticals have a better antitumor effect than drug treatment alone. The delivery of miRNA-7-5p in conjunction with a medication significantly reduced the ability of the treated cells to survive. Cancer cells treated with miR-7-5p have less chemoresistance. The gathered information shows a connection between the activation of MDR genes and the low intracellular level of miR-7-5p. In GB- (A172 and T98G), cervical-carcinoma- (HeLa), and PTC-derived (TPC-1) cells, it was demonstrated that a reconstitution of the miR-7-5p level led to a decrease in the expression of the PGP, BCRP, and MRP1 and 6 encoding genes [68].

### 6.8. Hepatocellular Carcinoma Therapy

ABZ-loaded cubosomal dispersions were prepared using GMO and P407 as a stabilizer. Albendazole (ABZ) has been discovered to be a potent inhibitor of numerous cancers, including hepatocellular carcinoma (HCC), which is the largest cause of cancer-related deaths globally. However, ABZ has a low bioavailability, and the processes behind its anticancer activities may be more complex than tubulin inhibition. We looked at the effects of ABZ suspension (i.p. and p.o.) and ABZ-loaded cubosomes (LC) on diethylnitrosamine-induced HCC in mice. The average particle size of ABZ-loaded nanoparticles was 48.17 ± 0.65 nm, with a 93.26 ± 2.48% entrapment effectiveness. When comparing ABZ-loaded cubs to aqueous ABZ suspension, the absorption analysis demonstrated a two-fold improvement in relative bioavailability. Reduced oxidative stress, enhanced liver function, and a lower necro-inflammation score were also discovered. ABZ effectively suppressed CD34 tissue, CD309 mRNA, and VEGF expressions at the protein level. MMP-9 and CXCR4 levels were also lowered, indicating an antimetastatic effectiveness. As revealed by the increased p53 mRNA expression, a greater Bax/BCL-2 ratio, and active caspase-3, ABZ showed a considerablelevel of apoptotic activity. Interrupting the ERK1/2-HIF-1-p300/CREB connection may thus be beneficial [69].

For the sustained release of anticancer drugs, such as cisplatin and paclitaxel, cubosomes coated with a layer of poly-ε-lysine were used. The dispersions of these formulations were investigated by using differential scanning calorimetry and X-ray diffractograms, and they were photographed using a transmission electron microscope. The medicine is completely disseminated in cubosomes according to in vitro tests. In addition, the zeta potential, in vitro release, and entrapment efficiency were assessed. Cubosomes have been discovered to be beneficial in avoiding an initial burst of anticancer medications followed by a steady release of anticancer chemicals. Using the human hepatoma HepG2 cell line, the coated, uncoated, and blank cubosomes were determined to be non-toxic. Impedance measurements and fluorescence imaging validated the cubosomes’ therapeutic efficacy against HeLa cells. Furthermore, the impairment of HeLa cells was demonstrated by the reduction in impedance in cells treated with coated combinatorial cubosomes, as validated by fluorescent microscopy [70].

### 6.9. Brain Tumor Therapy

Monoolein cubosomes were used to incorporate the anticancer agent doxorubicin (DOX). T98G glioblastoma cells and MTS cell proliferation assay were used to investigate the in vitro cytotoxicity of DOX-loaded cubosomes. Free DOX decreased T98G cell proliferation after 24 h at a dosage of 100 mg/mL but there was no inhibitory action at lower doses. Even after 72 h of incubation, the non-doped cubosome had no cytotoxic impact at a dosage of 90 mg/mL, and roughly 87% of metabolically active cells were present. In addition, after 24 h of incubation with T98G cells, the cytotoxic effect of DOX-loaded cubosomes with a DOX concentration of 2.3 mg/mL revealed that 70% of the viable cells were still present. The cytotoxicity of DOX integrated into cubic nanoparticles at a dosage of 2.3 mg/mL was higher than that of direct DOX delivery, according to our findings. Furthermore, a lower dosage of DOX was required to limit cancer cell proliferation than when free DOX was used [71].

### 6.10. Breast Cancer Therapy

The toxicity of blank cubosomes and cubosomes modified with folic acid is examined using MCF-7 cells. Blank cubosomes with GMO concentrations ranging from 10 to 1000 μg/mL were added to cells and incubated for 24 h, while FA-modified nanoparticles were used with a concentration of 1: 10 (100 μL/mL of medium). The MTT experiment revealed that neither the blank cubs nor the FAcubs are harmful to MCF-7 cells. The cytotoxicity of etoposide (ETP)-included cubosomes was then assessed using the MTT assay on the MCF-7 human breast cancer cell line, which has high amounts of folate receptors. When compared with free medication, ETP-Cubs had a significantly higher cytotoxicity. In MCF-7 cell lines, the IC50 values of the three groups increased in the sequence ETP > ETP-Cubs > ETP-Cubs-FA. The etoposide-loaded blank cubosomes’ tumor-targeting ability in this study was primarily a result of their EPR effects, whereas the folic-acid-modified cubosomes actively targeted the tumor through an interaction between the folate and its receptor, which overexpressed on the surface of MCF-7 breast cancer cells, resulting in a high level of tumor uptake [72].

**Table 4:** In vitro cytotoxicity of anticancer cubosomes.

Tumor	Cubosomes	Assay	Cell Line	Result	Reference
Liver Cancer	5-FU-loaded cubosomes	MTT assay	Hep G2 cell line	Cytotoxicity of 5-FU-loadedcubosomes is much higher than free drug only.	[66]
	Cisplatin- and paclitaxel-loaded cubosomes	MTT assay	Hep G2 cell line	Cytotoxicity of the uncoated-drug-loaded cubosomeswas more than the coated ones, which may be attributed to the faster release of drugs in the case of the uncoated ones.	[70]
Colorectal Cancer	Cisplatin and metformin nanocubosomes	Sulfo-rhodamine B (SRB) assay	HCT-116	The harmful effects of the drug-loaded nanoparticles were validated by the fraction of the cell survival values, which were higher than the effects of the individual drug.	[65]
Breast Cancer	Folic-acid-modified etoposide cubosomes	MTT assay	MCF-7cell lines	When compared with free medication, ETP-Cubs showed a significant increase in cytotoxicity.	[72]
Brain Cancer	Cubosomes loaded with temozolomide (TMZ) and doxorubicin (DOX)	MTS cell proliferation assay	T98G GB-derived cell lines	The viability of A172 and T98G cells was significantly reduced after cells were transfected with miR-7-5p and then treated with TMZ.	[68]
	DOX-loaded cubosomes	MTS cell proliferation assay	T98G glioblastoma cells	DOX incorporated into cubic nanoparticles at a concentration of 2.3µg/mL exerted higher cytotoxicity than direct DOX delivery.	[71]
	AT101-loaded cubosomes	Colorimetric WST-1 assay	A172cell lines	Encapsulated AT101 exhibited stronger cytotoxic effects and more extensive rearrangement of actin fibers in GBM cells than free AT101.	[63]
Ovarian Cancer	Icariin cubosomes	MTT assay	SKOV-3 and Caov 3	Indicated the cytotoxic potential of ICA-Cubs stops cancer cells from multiplying.	[67]
Cervical Cancer	Cubosomes loaded with doxorubicin labeled with 177Lu	MTS assay	HeLa cells	The cytotoxicity enhancement became statistically significant only after shorter incubation times.	[73]

## 7. Miscellaneous Drug Delivery by Cubosomes

### 7.1. Ocular Applications

Many studies have been conducted recently in order to evaluate the efficiency of cubosomes for ocular drug delivery by utilizing their benefits, such as being biodegradable, able to encapsulate all three types of drug molecules, i.e., hydrophilic, hydrophobic, and amphiphilic, and their rendering of bioactive agents with targeted and controlled releases. Because they have a long residence time at the corneal surface, cubosomes proved to have a high ocular bioavailability with the incorporated drugs. GMO-containing cubosomes have mucoadhesive properties that are beneficial in enhancing corneal permeability, and they subsequently enhanced the bioavailability of drugs. Excellent results were obtained when cubosomes with topical ocular drug delivery systems were studied. Cubosomes loaded with dexamethasone are widely studied for their in vitro permeation through excised rabbit corneas. The apparent permeability coefficient was increased, while the precorneal residence time test and pharmacokinetic study of aqueous humor samples results showed that the preocular retention time is increased when compared with Dex-Na phosphate eye drop, and simultaneously there was an overall increase in dexamethasone concentration in the aqueous humor [74,75].

### 7.2. Dermatological Applications

Dermatological drugs are used to treat skin diseases, and in order to deliver drugs through the dermis, the stratum corneum acts as a front barrier because it is highly organized. It restricts the penetration of agents through the skin when applied topically. However, due to the unique structural configurations and properties of cubosomes, they provide the opportunity to act as a carrier for transdermal drug delivery. GMO-containing cubosomes can be effectively used in the mucosal and topical delivery of drugs due to the bioadhesive properties of GMO on the stratum corneum. Another promising topical application of cubosomes is vaccination through trans-cutaneous immunization (TCI). In this technique, microneedles (MNs) and cubosomes have been used successfully as a synergistic method for the delivery of vaccines via skin. It is revealed that the microneedle enhances the permeation of the peptide mixture in water through the skin layers, and cubosomes with peptide showed longer retention within the skin. Subsequently, the use of combined microneedle and cubosome approaches were proved to be anefficient system for the local delivery of antigens to the targeted cells in the skin [76,77].

### 7.3. Oral Delivery

Although oral drug delivery is most convenient and long-used method of drug delivery, it usually poses complications for the delivery of poorly water-soluble drugs—drugs that are poorly absorbed and with a large molecular weight. Cubosomes assist the absorption of orally administered drugs, possibly because of their interactions with the intestinal cell membrane or their induction of physiological secretions during lipid digestion in GI tract. It can be because of the bioadhesive properties of cubosomes that these agents are effectively administered orally. A study was conducted to orally administer cubosomes loaded with insulin to fasted streptozotocin-induced diabetic rats. In this study, water, emulsifier, and GMO were microfluidized at 80 °C and then cooled at room temperature. In order to keep insulin stable under harsh conditions, the formulation was prepared at room temperature and large aggregates were collected. The mucoadhesive nature of GMO and the biocompatibility of cubosomes, as well as the reproducible hypoglycemic effect and an enhanced adsorption on the intestinal epithelia, are achieved. In addition, cubosomes can be a vital vehicle for the oral delivery of poorly water-soluble agents. The oral delivery of drugs is incorporated in the solubilized form and within the lipid bilayer portion of their structure, which prevents drug precipitation in the gastro-intestinal tract and enhances intestinal adsorption due to the mucoadhesive nature of GMO [78].

## 8. Drugs Embedded in Cubosomes

As described earlier regarding the various applications of cubosomes in pharmaceutical drug delivery, Table 5 describes the list of drugs that researchers attached or incorporated into cubosomes for their effective and targeted delivery.

## 9. Challenges and Future Perspectives

Apart from all of these benefits and recent breakthroughs, there are some issues and areas of worry that need to be addressed. The most significant disadvantage to overcome is that large-scale production is sometimes difficult because of high viscosity, and there is a low trapping of water-soluble medicines due to the presence of huge volumes of water inside cubosomes [85]. The surface modification of cubosomes with certain polymers (such as poly-lysine) can minimize the burst release of hydrophilic medicines, such as cisplatin, in this regard. Unfortunately, there have been no convincing investigations on cubosomal oxaliplatin and carboplatin formulations as of yet. As a result, more research is needed to determine the anticancer effects of these formulations in vivo and in vitro [86].

They are also appealing nanovehicles for loading and delivering proteins and peptides but the reported studies are still at a foundational level, and the different aspects of the structural and morphological characteristics of these soft nanocarriers, as well as their loading capacity and release, should be addressed. Blood compatibility should be considered early in the development of cubosome-based intravenous nanomedicines in the future. In addition, little is known about their stability in biological fluids and the biological variables that regulate drug release from cubosomes; the structural transformations upon contact with biological fluids, such as plasma; interactions with cell membranes; and their infusion-related reactions, to mention a few. The use of cubosomes for intravenous drug delivery is ambitious; nevertheless, these nanocarriers may find expedited uses for the delivery of poorly water-soluble medicines via oral, ophthalmic, and topical routes, providing a cost-effective option in formulation science [87,88].

Cubosomes’ pharmaceutical uses are rapidly expanding; yet, the results presented in this study revealed that much more research is needed to fully comprehend the key features of its drug transportation capabilities, NP biological tissue interactions, and in vivo biodistribution. Such a lag in the pre-clinical development of cubosomes is reflected in the lack of globally commercialized products. While various liposome formulations are on the market or in clinical studies (Doxil^®^ and Lipo-Dox^®^, to name a few), the pharmaceutical regulatory approval of non-lamellar LLC NPs has yet to be granted. The creation of multifunctional nanoplatforms has piqued interest as a promising way to integrating drug administration and imaging in the endeavor to efficiently create bioactive cubosomes and adapt them into clinical practice [89,90].

## 10. Conclusions

Cubosomes are a type of lipid-based nanovesicle that is distinguished by the liquid crystalline form of their nanostructure. They are made from amphiphilic lipids that self-assemble into cubosomes in the presence of a stabilizer. Several recent studies have demonstrated their potential as a new medication delivery mechanism. Cubosomes have been approved as an effective ocular drug delivery system, which has increased ocular residence time, bioavailability, and reduced eye irritation. Cubosomes were shown to be beneficial in increasing the absorption of weakly water-soluble medications, protecting the liable drug from enzymatic destruction, and delivering targeted drugs in an oral application. They offer a promising method for efficient transdermal medication administration, with improved skin penetration and less irritation. Cubosomes were used to administer anticancer medications, which resulted in fewer major side effects from the chemotherapeutic treatments and a more targeted drug delivery. Cubosomes’ pharmaceutical uses are rapidly expanding; yet, the results presented in this study revealed that much more research is needed to fully comprehend the key features of its drug transportation capabilities, NP biological tissue interactions, and in vivo biodistribution.

## Figures and Tables

**Table 1:** Stabilizing agents and lipids used in preparation of cubosomes.

Lipids	Stabilizer	Reference
Monoolein	Pluronic F127	[29]
Phytantriol	Pluronic F127	[30]
Monoelaidin	Pluronic F127	[31]
β-XP (1-*O*-phytanyl-β-d-xyloside)	Pluronic F127	[32]
Monoolein or phytantriol	Pluronic F108	[33]
Phytantriol	Myrj 59	[34]
Monoolein	Modified starch	[27]
Sodium octyl sulfate (SCS)	Arginine-based cationicsurfactant	[35]
Monoolein	Laponite XLG	[36]

**Table 2:** Comparison of techniques.

Techniques	Benefit	Drawback
Top-down approach	Formulation has stability against aggregation for up to one year.	It requires high energy input to disperse the aggregates into cubosomes.
Bottom-up approach	It requires low energy input; thus, it can be safely used with temperature-sensitive agents.	Preferable for only thermo-sensitive reactants, and preparations are stable for less time.
Spray-drying method	The technique is a highly versatile, cheap, and scalable method. It is well-suited for drying labile products, such as vaccines and proteins.	The mixture was difficult to spray-dry as a cubic phase is immediately formed upon hydration of monoolein.
Solvent evaporation method	Cubosomes formed using solvent evaporation approach are smaller, with higher physical stability.	High polydispersity of particle sizes is reported due to large-scale mixing of ethanol and water.

**Table 3:** Physical–chemical characterization of drugs-loaded cubosomes.

Cubosomes	Composition of Cubosomes	Particle Size (nm)	Zeta Potential (mV)	Encapsulation Efficiency (EE%)	Polydispersity Index (PDI)	Reference
Cubosomes loaded with antimicrobial peptides	GMO, Poloxamer 407, antimicrobial peptide LL-37	191.7 ± 12.0	−24.8 ± 3.4	60.0	0.05 ± 0.02	[49]
Latanoprost-loaded phytantriolcubosomes	Phytantriol, F127, latanoprost	209.3 ± 5.1	−24.5 ± 0.6	94.00 ± 3.16	0.11 ± 0.01	[50]
Inhalable-bedaquiline-loaded cubosomes	-	150.2 ± 5.1	35.4 ± 2.3	51.85 ± 4.83	0.24 ± 0.02	[51]
Norfloxacin-loaded nanocubosomes	GMO, F108, Cremophor	216.8 ± 2.5	−41.2 ± 2.3	94.3 ± 1.4	0.3 ± 0.0	[52]
Ketoconazole-loaded cubosomes	GMO, Poloxamer 407, PVA	381 ± 2.082	-	2.22 ± 1.08	0.918 ± 0.0	[53]
Chitosan-modified ginseng stem–leaf-saponins-encapsulated cubosomes	GMO, Poloxamer 407, chitosan, ginseng stem–leaf saponins	204.93 ± 5.80	29.90 ± 0.551	60.47 ± 4.72	0.160 ± 0.015	[54]
Dexamethasone-loaded cubosomes	GMO, Poloxamer 407, oleic acid	250.40 nm	−36.10 ± 2.56	93.8	-	[55]
Duloxetine-HCL-loaded cubosomalgel	GMO, glycerol tripalmitate, Pluronic F68 and F127	145.8 ± 4.8	1.6 ± 0.21	98.57 ± 0.51	1 ± 0.1	[56]

**Table 5:** Drugs embedded in cubosomes.

Drugs	Objective of Study	Outcome of Study	Reference
Docetaxel	Synthesis and evaluation of controlled release of cubosomes incorporated with docetaxel as thermo-sensitive depot.	The depot offered gradual drug release, preparation wasfree-flowing at room temperature, and changed to the depot at bodytemperature.	[79]
Antimicrobial peptide LL-37	The antimicrobial potential of cubosomal LL-37 was evaluated using in vitro andexvivo skin irritation models.	The formulation provides superior protection to LL-37 against enzymatic degradation and significant bactericidal effects, and ensures a controlled release. Cubic nanoparticles reduce skin irritation due to LL-37.	[49]
Ketorolac	Monoolein and poloxamer cubicnanoparticles for ocular delivery of ketorolac.	Optimized cubosomes loaded with Ketorolac provided transcorneal permeation and retention.	[80]
Indomethacin	Evaluation of Indomethacin-fabricated cubosomes for anti-inflammatoryactivity.	Homogenized-monoolein- andpoloxamer-containing-cubosomes prolonged the delivery of lipophilic drugthrough the skin.	[23]
Flurbiprofen (FB)	NSAID used for treatment of ocular inflammation.	The formulation expressed less ocular irritation and enhanced trans-corneal permeation of FB.	[80]
Erythromycin	Treatment and prevention of several types of acne as a result of its bacteriostaticactivity againstPropionibacterium acnes.	The formulation prevents the acne due to the topical application of erythromycin impregnated with cubosomes.	[81]
Insulin	Tested against the C-Type-1-diabetic-induced rat (insulin-dependent diabetes).	Cubosomes provide shield to insulin against proteolysis. It is found to be stable at normal temperature and controlled the hyperglycemia in a reproducible manner.	[82]
20(S)protopanaxadiol (PPD)	To improve the bioavailability of antitumor drug.	Cubosomes enhanced the oral bioavailability of PPD as a result of enhanced absorption of sparinglywater-soluble drug.	[83]
Dacarbazine	To reduce the side effectsagainst melanoma.	Dacarbazinedelivered through cubosomes decreases the side effects of intravenous delivery. It also enhanced drug efficacy, safety, and shelf life.	[84]

## Data Availability

Not applicable.
